# A New Proof of the Expected Frequency Spectrum under the Standard Neutral Model

**DOI:** 10.1371/journal.pone.0118087

**Published:** 2015-07-21

**Authors:** Richard R. Hudson

**Affiliations:** Department of Ecology and Evolution, University of Chicago, Chicago, IL, United States of America; UC Santa Barbara, UNITED STATES

## Abstract

The sample frequency spectrum is an informative and frequently employed approach for summarizing DNA variation data. Under the standard neutral model the expectation of the sample frequency spectrum has been derived by at least two distinct approaches. One relies on using results from diffusion approximations to the Wright-Fisher Model. The other is based on Pólya urn models that correspond to the standard coalescent model. A new proof of the expected frequency spectrum is presented here. It is a proof by induction and does not require diffusion results and does not require the somewhat complex sums and combinatorics of the derivations based on urn models.

## Introduction

A useful summary description of DNA sequence variation in samples from a population is the sample frequency spectrum. The sample frequency spectrum for a sample of size *n*, is a vector, $\{s_{i}^{\left(n\right)}\},i=1,\ldots ,n-1$, where $s_{i}^{\left(n\right)}$ is the number of polymorphic sites at which there are *i* copies of the mutant allele in the sample. This sample frequency spectrum has been the basis for numerous estimators and test statistics for analyzing population genetics data. (See for example section 6.4 of Charlesworth and Charlesworth [1].) Under the standard infinite-sites neutral model with constant diploid population size, *N*, it is well known that:

$$\begin{array}{c} \text{E}\left(s_{i}^{\left(n\right)}\right)=\frac{\theta }{i},\;i=1,\ldots ,n-1, \end{array}$$ (1)

where E() denotes expectation and *θ* = 4*Nu*, where *u* is the neutral mutation rate (Fu [2]). A polymorphic site at which there are *i* copies of the mutant allele will be referred to as a polymorphism of size *i*, and the mutation that generated that polymorphisms will be referred to as a size *i* mutation. Under the infinite-sites model of mutation, every mutation is assumed to occur at a site not previously mutated, in which case the number of polymorphisms equals the number of mutations in the genealogy of the sample. Thus $s_{i}^{\left(n\right)}$ denotes both the number of size *i* polymorphisms in the sample and the number of size *i* mutations in the genealogy of the sample. Although Eq (1) is written with an “=” sign, it is actually a limiting result for large *N* and small *n*. Eq (1) has been derived previously in several ways. Ewens [3] and Tajima [4] obtained essentially this equation based on diffusion methods. Fu [2] derived Eq (1) via somewhat complex sums and combinatorics based on Pólya urn models. Griffiths and Tavaré [5] obtained a generalization of Eq (1) also based, at least in part, on the urn model approach. In this note, we present a new proof of Eq (1) using induction that avoids these complications.

Before presenting our new proof of Eq (1), it is useful to review some elementary properties of the standard coalescent for a sample of size *n*. We assume the standard coalescent model with constant population throughout this note. An excellent description of this model and its properties are provided by Wakeley [6]. First, the process is usually thought of as a backward looking process, where the lineages leading to the sample, are traced backwards in time until common ancestors are encountered. Initially, one traces *n* distinct ancestral lineages back in time until a random pair of lineages have a common ancestor. We say that a coalescent event has occurred at this time, as the two lineages with a common ancestor are merged into a single ancestral lineage. The time interval from the present back to this coalescent event, denoted *t*_*n*_, has an exponential distribution with mean equal to $\frac{4N}{n(n-1)}$. The process is followed further back in time, now following *n* − 1 lineages. (These *n* − 1 lineages consist of the *n* − 2 lineages not involved in the first coalescent event and the single lineage ancestral to the two lineages that have coalesced.) After a random interval of time, another coalescent event occurs, at which a random pair of the *n* − 1 lineages coalesce. This process is continued until the number of ancestral lineages is reduced to one, at which point one has arrived at the most recent common ancestor of the sample. The time intervals between coalescent events are independent exponentially distributed random variables. The mean length of the interval during which there are *i* distinct ancestral lineages is given by:

$$\begin{array}{c} \text{E}\left(t_{i}\right)=\frac{4N}{i(i-1)},\;\;\;i=2,\ldots ,n. \end{array}$$ (2)

It follows from Eq (2) that the expected number of generations back to the most recent common ancestor of the sample, *t*_MRCA_, is

$$\begin{array}{c} \text{E}\left(t_{\text{MRCA}}\right)=\text{E}(\sum_{i=2}^{n}t_{i})=4N\left(n-1\right)/n. \end{array}$$ (3)

Under the infinite-sites model of mutation, it is assumed that the number of mutations that occur along a lineage is Poisson distributed with mean *ut*, where *t* is the length of the lineage in generations. From Eq (3) it follows, under the infinite-sites model, that the average number of mutations that distinguish a sampled allele from the most recent ancestor of a sample is *u*E(*t*_MRCA_) = *θ*(*n* − 1)/*n*. Similarly, the expected total number of polymorphic sites, denoted *S* ^(*n*)^, is *u* times the expected sum of lengths of the branches of the gene tree. Then, using Eq (2) we have:

$$\begin{array}{c} \text{E}\left(S^{\left(n\right)}\right)=u\sum_{i=2}^{n}i\text{E}\left(t_{i}\right)=\theta \sum_{i=1}^{n-1}1/i. \end{array}$$ (4)

Note that $S^{\left(n\right)}=\sum_{i=1}^{n-1}s_{i}^{\left(n\right)}$.

**Fig 1:**
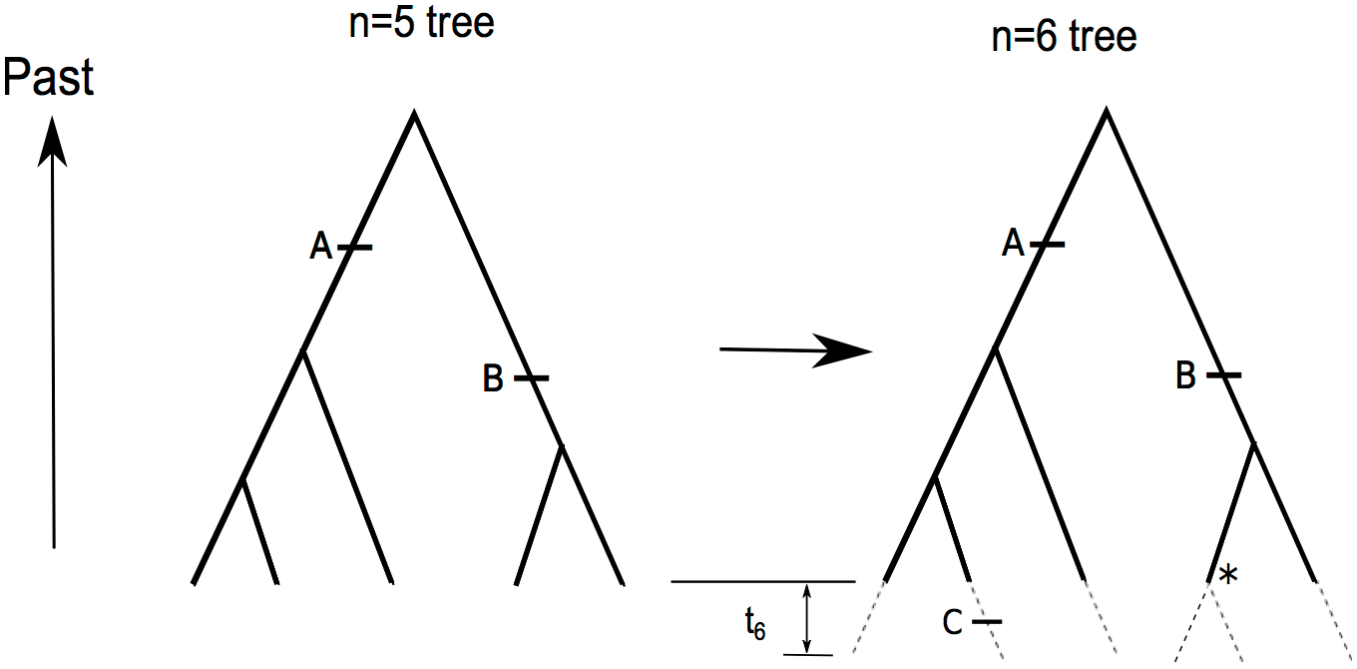
Generating a sample size 6 tree by extending tip branches of a sample size 5 tree. The short horizontal bars indicate mutations, and the asterisk indicates the branch which is split as described in the main text. The dashed lines indicate the branch extensions also described in the main text. The mutation labeled A is a size 3 mutation on both the (*n* = 5)-tree and the (*n* = 6)-tree. The mutation labeled B is a size 2 mutation on the (*n* = 5)-tree, but a size 3 mutation on the (*n* = 6)-tree. The mutation labeled C is a mutation on a branch extension and is a size 1 mutation on the (*n* = 6)-tree.

Fig 1 shows two example coalescent trees produced by this coalescent process. The tree on the left has two mutations on it, one of size three and one of size two. The tree on the right has an additional mutation of size one.

## Proof that $\text{E}(s_{i}^{\left(n\right)})=\frac{\theta }{i}$

With these preliminaries out of the way, we now present our proof of Eq (1). For *n* = 2, all polymorphisms are of size 1, and so our result for expectation of *S* ^(*n*)^ applies directly to give $\text{E}(s_{1}^{\left(2\right)})$. That is, for *n* = 2, applying (4) we find:

$$\begin{array}{c} \text{E}\left(s_{1}^{\left(2\right)}\right)=\text{E}\left(S^{\left(2\right)}\right)=\theta , \end{array}$$ (5)

and thus Eq (1) holds for *n* = 2.

We now show that if Eq (1) holds for *n*, that it is also true for *n*+1. To do this, we note that one can generate a sample size *n*+1 gene tree with its mutations by first generating a sample size n tree with its mutations and then extending it as follows. One picks a random tip of the size *n* tree, splits that tip, and then extends all the tips by a random time. The random time added to each of the tips is exponentially distributed with mean $\frac{4N}{n(n+1)}$. That is, the time extension is distributed like *t*_*n*+1_. Then each of the *n*+1 lineage extensions experience mutation according to the infinite-sites model. This method of getting a sample size *n*+1 tree is shown in Fig 1. This exact approach was used by Fu and Li [7] to derive other properties of this model. (This approach to generating a sample size *n*+1 tree from a sample size *n* tree, is not to be confused with idea of sampling an additional allele from a population after sampling *n* alleles, and then examining how it coalesces into a lineage of the sample size *n* tree.)

With this method of generating a sample of size *n*+1, we can relate the polymorphisms of size *i* in a sample of size *n*+1 to the polymorphisms of size *i* and *i* − 1 of the sample of size *n* and its tree. The tree for the sample of size *n* and its mutations, we call the *n*-tree, and the extended tree with its mutations the (*n*+1)-tree. We refer to the tip of the *n*-tree that is split as the *-tip, and the mutations on the lineage from that tip back to the most recent common ancestor of the n-tree we call *-mutations. If a *-mutation is of size *i* in the n-tree, then it will be of size *i*+1 in the (n+1)-tree. If a mutation of size *i* in the n-tree is not a *-mutation, then it will also be of size *i* in the (*n*+1)-tree. An illustrative example is shown in Fig 1. We designate the number of size *i* mutations that are *-mutations by $s_{i,*}^{\left(n\right)}$.

From the above considerations, it follows that:

$$\begin{array}{c} s_{i}^{\left(n+1\right)}=s_{i}^{\left(n\right)}-s_{i,*}^{\left(n\right)}+s_{i-1,*}^{\left(n\right)},\;\;i=2,\ldots ,n, \end{array}$$ (6)

providing that we define $s_{n}^{\left(n\right)}$ and $s_{n,*}^{\left(n\right)}$ to be zero. Note that all mutations of size 2 and larger in the (*n*+1)-tree are due to mutations that are already present in the *n*-tree. The case of *i* = 1, is special, and is treated later. Taking expectations of both sides of (6), and using our assumption that (1) holds for sample size *n*, we find:

$$\begin{array}{c} \text{E}\left(s_{i}^{\left(n+1\right)}\right)=\frac{\theta }{i}-\text{E}\left(s_{i,*}^{\left(n\right)}\right)+\text{E}\left(s_{i-1,*}^{\left(n\right)}\right),\;\;i=2,\ldots ,n. \end{array}$$ (7)

To obtain $\text{E}(s_{i,*}^{\left(n\right)})$, we label the size i mutations of the n-tree from 1 to $s_{i}^{\left(n\right)}$ and write $s_{i,*}^{\left(n\right)}$ as the sum of indicator variables:

$$\begin{array}{c} s_{i,*}^{\left(n\right)}=\sum_{j=1}^{s_{i}^{\left(n\right)}}X_{j}, \end{array}$$ (8)

where *X*_*j*_ is one if the size *i* mutation labelled *j* is a *-mutation, i.e., if the *-tip is a descendant of the size *i* mutation labelled *j*, and zero otherwise. The probability that a specific size *i* mutation is a *-mutation, is just $\frac{i}{n}$, since the *-tip is a random tip, and there are *i* descendant tips of a size *i* mutation. It follows that the expectation of *X*_*j*_ is $\frac{i}{n}$ and that

$$\begin{array}{c} \text{E}\left(s_{i,*}^{\left(n\right)}\right)=\text{E}\left(s_{i}^{\left(n\right)}\right)\text{E}\left(X_{j}\right)=\frac{\theta }{i}\frac{i}{n}=\frac{\theta }{n}, \end{array}$$ (9)

which, surprisingly, does not depend on *i*. This means that the last two terms on the right hand side of Eq (7) cancel each other, and that Eq (1) holds for sample size *n*+1 for *i* = 2, …, *n*.

We now consider size one mutations. From Eq (4) we know E(*S* ^(*n*+1)^), and from the result just obtained, we know the expected number of mutations with size 2 or greater, so we can obtain the expected number of size one mutations by substraction:

$$\begin{array}{c} \text{E}\left(s_{1}^{\left(n+1\right)}\right)=\text{E}\left(S^{\left(n+1\right)}\right)-\sum_{i=2}^{n}\frac{\theta }{i}=\theta . \end{array}$$ (10)

Alternatively, we could obtain $\text{E}(s_{1}^{\left(n+1\right)})$ by consider mutations that happen on the extensions of the tip branches of the (*n*+1)-tree. Every such mutation results in a size one mutation in the (*n*+1)-tree. There are (*n*+1) such tip branches that are extended on average $\frac{4N}{(n+1)n}$ generations, so an average of $u(n+1)\frac{4N}{(n+1)n}=\frac{\theta }{n}$ new mutations are added to the tree. Thus for *i* = 1, we have:

$$\begin{array}{c} s_{1}^{\left(n+1\right)}=s_{1}^{\left(n\right)}-s_{1,*}^{\left(n\right)}+\frac{\theta }{n}. \end{array}$$ (11)

Taking expectations, we see that the last two terms cancel again, and that Eq (1) holds for *i* = 1. This completes the proof.
